# Joint Methodology Based on Optical Densitometry and Dynamic Light Scattering for Liver Function Assessment

**DOI:** 10.3390/diagnostics13071269

**Published:** 2023-03-28

**Authors:** Elina Karseeva, Ilya Kolokolnikov, Ekaterina Medvedeva, Elena Savchenko

**Affiliations:** 1 Higher School of Applied Physics and Space Technologies, Institute of Electronics and Telecommunications, Peter the Great St. Petersburg Polytechnic University, Saint Petersburg 195251, Russia; 2 Computer Information Systems Department, International University of Kyrgyzstan, Bishkek 720010, Kyrgyzstan

**Keywords:** blood flow, correlation analysis, scattering, sensors, microcirculation, optical densitometry, liver, dynamic light scattering, indocyanine green

## Abstract

A pressing health problem, both in clinical and socio-economic terms, is the increase in the number of patients with liver damage caused by viral diseases (hepatitis), cancer, toxicological damage, or metabolic disorders. Liver function assessment is a complex task, for which various existing diagnostic methods are used. Unfortunately, they all have several limitations which frequently make prompt and accurate diagnosis impossible. The high level of disability and mortality caused by liver diseases makes the development of new liver diagnostic methods very urgent. In this paper, we describe a new joint methodology for studying liver function based on optical densitometry and dynamic light scattering. This will help to diagnose and predict the dynamics of liver function during treatment with greater efficiency, due to including in consideration the individual characteristics of the cardiovascular system and tissue metabolism. In this paper, we present a laboratory model of a combined sensor for optical densitometry and dynamic light scattering. We also developed special software for controlling the sensor and processing the recorded data. Modeling experiments and physical medical studies were carried out to adjust and calibrate the sensor and software. We also assessed the sensor resolution when registering the concentration of dye in the human body and the minimum measured flow rate.

## 1. Introduction

One of the topical issues in modern hepatology is the assessment of the liver hepatodepression level. Liver-associated pathologies are among the most common chronic pathologies in most developed countries of the world [1]. With proper disease control, concomitant liver damage can be minimized, while the uncontrolled development of a disease can lead to liver cirrhosis and consequentially to liver resection with subsequent transplantation. To monitor the liver state clinicians can use quantitative assessment of its functional reserves [2,3,4].

Traditionally, this procedure is carried out by blood analysis, although this gives insufficient data about liver function. Modern methods of liver-state detection include measurement of its elimination function with a special dye injected into the bloodstream. As a dye, indocyanine green (ICG) is usually used [5]. By drawing a number of blood samples after the dye injection, the doctor measures the dye’s concentration dependence on the time after injection. This dependence is associated with liver function [6]. Although this method is frequently used, it has many disadvantages including high hygiene demands, a long time required for analysis, and the necessity for sophisticated spectrometric equipment, making it ineffective for prompt medical decisions. To avoid these limitations, we can use non-invasive monitoring based on optical pulse densitometry [7]. In this method, light is transmittedthrough the patient’s tissue, at a wavelength selected based on the absorption spectrum of the dye. Its absorption in peripheral vessels in vivo is associated with dye concentration in the blood. Sensors based on the described method, the so-called clearance test, are already used in modern medical diagnostics (e.g., Sensor LiMON by Pulsion Medical Systems AG) [8]. Such sensors are non-invasive and allow results in about 15 min after the start of the measurement. However, they regularly demonstrate imperfection in terms of reliability due to the strong influence of hand tremors, patient movement, and changes in pulse and breathing rate causing errors in data processing within the device [9,10]. In addition, the blood vessels’ status can influence the final result [11]. It is important to consider that the elimination of indocyanine green can depend on the microcirculation system state, which is currently not taken into account during regular tests.

Processing problems are related to incorrect determination of the dye injection time and the first recirculation peak position. These problems are obtained in more than 10% of the measurements, leading to repeated testing in those cases. Considering the high price of dye used and the need for a few hours’ gap between tests [12], it is highly desirable to minimize the rate of processing error.

Differences in the microcirculation system of each patient, in particular, make it hard to analyze real liver function based on measured quantitative value by the dynamic clearance test (mainly using as a parameter the elimination time of exogenous substances by the liver). Microcirculation disorders play a very important role in the pathogenesis of many diseases; therefore, objective registration of microcirculatory disorders can provide enough data for a deeper understanding of various symptoms’ origins. Changes in the blood microcirculation system closely correlate with changes in central hemodynamics, allowing the use of microcirculation parameters as prognostic and diagnostic criteria for assessing the general physical condition and health of patients [13,14]. In this regard, research aimed at improving the efficiency of diagnosing microcirculatory disorders is extremely relevant.

It seems that assessing the microcirculation state together with the clearance test will make it possible to diagnose and predict more effectively the dynamics of changes in liver function during treatment. In this research, a joint methodology is proposed based on optical densitometry and dynamic light scattering. It has been developed for simultaneous measurement of ICG plasma elimination rate and blood flow velocity in the microcirculatory bloodstream, to allow complex analysis of liver function considering possible microcirculation disorders.

## 2. Materials and Methods

**Figure 1:**
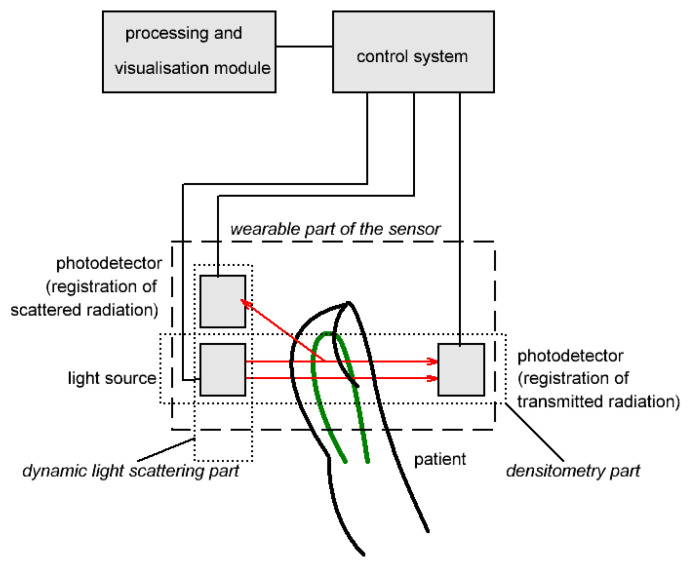
Design of the combined densitometry–dynamic light-scattering setup for liver function assessment. Arrows show transmitted and scattered light.

The combined experimental setup for liver function evaluation is presented in Figure 1. It includes two main parts: a densitometry sensing system for evaluation of ICG plasma elimination rate and a dynamic light-scattering sensor for measurement of blood flow velocity.

The wearable part of the finger-piece optical sensor contains a light source and two photodetectors to detect transmitted and scattered radiation. The combined wearable part of the sensor allows simultaneous detection of both described parameters. The control system manages the power supply for the light source, collects data from photodetectors, and transmits it to the output device. The output device performs signal processing and visualization of results.

The following paragraphs give detailed explanations of the two proposed sensor parts and methods on which they are based.

### 2.1. Optical Densitometry

As described above, optical densitometry is based on the detection of light, transmitted through patient’s tissue (e.g., finger). The detected light intensity varies depending on the concentration of the absorbent in the examined tissue. In general, the relation between transmitted and incident light contains information about the investigated matter. The ratio of transmitted light intensity to incident light intensity can generally be described by the Beer–Lambert–Bouguer law:

(1) $$I_{T}=I_{0}\cdot 10^{-\alpha x}$$

where $I_{T}$ is the transmitted light intensity, $I_{0}$ is the incident light intensity, α is the decimal coefficient of absorption, or absorptivity for short, and *x* is the path of the light beam in the medium under study. Absorptivity is proportional to concentration of the absorbing agent, and the whole expression in decimal power is named absorbance:

(2) $$A=\alpha x=\varepsilon cx=-lg\left(\frac{I_{T}}{I_{0}}\right)$$

where *A* is absorbance, *ε* is the decimal extinction coefficient. Accordingly, the current solution concentration can be calculated from the transmitted light intensity when concentration is zero ($I_{0}$), and measurement of current transmitted light intensity ($I_{T}$), if extinction coefficient *ε* and path length *x* are known:

(3) $$c=\frac{1}{\varepsilon x}\cdot lg\left(\frac{I_{0}}{I_{T}}\right)$$

**Figure 2:**
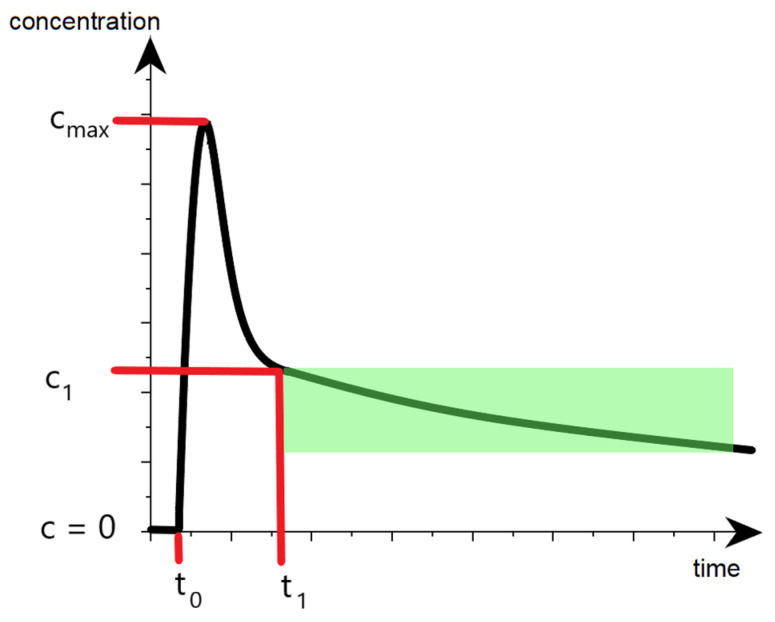
Illustration of diagnostical colorant concentration dynamic during the clearance test. t_0_ is the time when the first wave of injected colorant reached the volume under observation, c_max_ stands for concentration peak detected during the recirculation process. The green area is used for experimental *PDR* calculation.

Diagnostical colorant concentration dynamics in the patient’s blood during liver function evaluation testing can be approximated as an exponential function of time:

(4) $$c=c_{1}\cdot \exp\left(-0.01\cdot PDR\cdot \left[t-t_{1}\right]\right)$$

where $c_{1}$ is the dye concentration after its injection and even distribution in the blood plasma, *PDR* (plasma disappearance rate) is a parameter used to describe the rate at which the dye is removed from the blood by the liver, measured in %/min, *t* is time from the test start, measured in minutes, and *t*_1_ is the time when the blood recirculation process is considered as ended. The approximate dynamic of dye concentration during the clearance test is presented in Figure 2.

Skipping the details of data calibration, to connect the transmitted light intensity measurements and plasma disappearance rate calculations we should substitute concentration in Equation (4) with the intensity of the transmitted light as in Equation (3), which leads us to the following:

(5) $$\frac{1}{\varepsilon x}\cdot lg\left(\frac{I_{0}}{I_{T}\left(t\right)}\right)=\frac{1}{\varepsilon x}\cdot lg\left(\frac{I_{0}}{I_{T}\left(t_{1}\right)}\right)\cdot \exp\left(-0.01\cdot PDR\cdot \left[t-t_{1}\right]\right)$$

(6) ft=PDR·t−t1=−lnlgI0ITtlgI0ITt1·100%

$I_{T}\left(t\right)$ is an experimental set of transmitted light intensity points. $f\left(t\right)$ least squares linearization allows calculation of the *PDR* as a slope of this function.

It is obvious that the error of *PDR* determination depends completely on concentration measurement error. The concentration of the analyte in the test volume is determined in the experiment by measuring the intensity of the light that has passed through the sample and the intensity of the light that has passed through the control sample that does not contain the analyte, so Equation (3) can be written as:

(7) $$c=\frac{ln\left(10\right)}{\varepsilon \cdot z}\cdot \left(ln\left(I_{0}\right)-ln\left(I\right)\right)$$

Then, standard deviation of the concentration can be written as follows:

(8) $$∆c=\frac{ln\left(10\right)}{\varepsilon \cdot z}\cdot \sqrt{{\left(∆ln\left(I_{0}\right)\right)}^{2}+{\left(∆ln\left(I\right)\right)}^{2}}\underset{∆I\ll I}{\to }\frac{ln\left(10\right)}{\varepsilon \cdot z}\cdot \sqrt{{\left(\frac{∆I_{0}}{I_{0}}\right)}^{2}+{\left(\frac{∆I}{I}\right)}^{2}}$$

**Figure 3:**
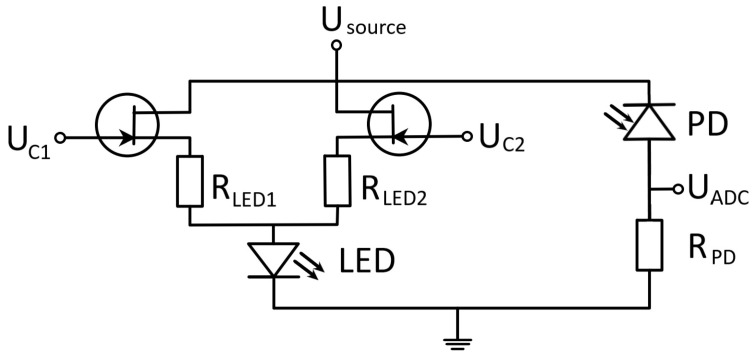
Schematic of electronic chain of the sensor. LED—light emitting diode, PD—photodiode, U_source_—source voltage, U_c1_ and U_c2_—control voltages for LED power supply control, U_adc_—ADC input voltage, R_led1_, R_led2_, and R_pd_—load resistors.

In the designed setup (Figure 3) the transmitted light intensity is measured by photodiode and is proportional to the photocurrent. The intensity of light reaching the photodiode can be measured by voltage on the load resistor.

Therefore, relative deviation of the intensity equals relative deviation of the detected voltage:

(9) $$I=s\cdot i=s\cdot \frac{u}{R},\;\;\;\;\;\;\;\;\frac{∆I}{I}=\frac{∆u}{u}\cdot \frac{s\cdot R}{R\cdot s}=\frac{∆u}{u}$$

where *s* is photodiode sensitivity, and *R* is load resistance. We can write the expression for standard deviation of the concentration as a function of voltage:

(10) $$∆c=\frac{ln\left(10\right)}{\varepsilon \cdot z}\cdot \sqrt{{\left(\frac{∆u}{u_{0}}\right)}^{2}+{\left(\frac{∆u}{u}\right)}^{2}}=\frac{ln\left(10\right)\cdot ∆u}{\varepsilon \cdot z}\sqrt{{\left(u_{0}\right)}^{-2}+{\left(u\right)}^{-2}}$$

It can be seen that the standard deviation of colorant concentration depends not only on standard deviation of the voltage but also on the reference measurements. Voltage on the load resistance is proportional to the intensity of the transmitted light; we can also write the voltage as a function of concentration:

(11) $$u=u_{0}\cdot 10^{-A}=u_{0}\cdot exp\left(-\ln\left(10\right)\cdot \varepsilon \cdot c\cdot z\right)$$

which gives us the standard deviation of the concentration as a function of the concentration:

(12) $$∆c=\frac{ln\left(10\right)\cdot ∆u}{\varepsilon \cdot z\cdot u_{0}}\sqrt{1+{\left(exp\left(-\ln\left(10\right)\cdot \varepsilon \cdot c\cdot z\right)\right)}^{-2}}$$

**Figure 4:**
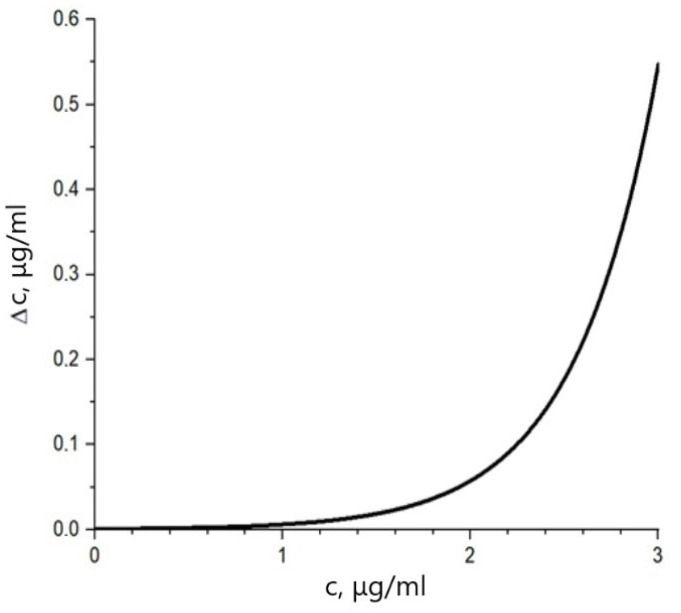
Standard deviation of measured dye concentration depending on the real dye concentration in tissue.

The standard deviation of measured concentration depending on the real dye concentration in the tissue can be visualized as an exponential curve (Figure 4). The dependence is constructed within the range of regular dye concentrations used in medical diagnostics [2,3,4,6,7,8,9]. The curve given by Equation (12) shows that the concentration determination error rises dramatically with the increase of colorant concentration. This rise is counterintuitive but is also correlated to the nonlinearity of the relationship between concentration and absorbance in the Beer–Lambert–Bouguer law correction for high concentration. The meaning of the curve in Figure 3 in the existence of an optimum range of concentrations to be used in medical practice. It is necessary to increase the colorant concentration to be able to observe the colorant absorbance against the background of body absorption. However, there is an upper limit to the reliable concentration measurement. Calibration curves for the Beer–Lambert–Bouguer law correction can be used to increase the method’s reliability for high concentrations [15,16,17].

**Figure 5:**
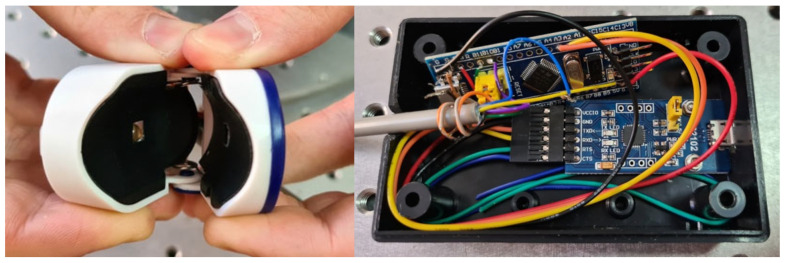
Wearable part of the sensor (**left**) and its control module (**right**).

Images of the wearable part of the sensor and its control module are presented in Figure 5. The sensor controller is STM32 f103c8t6 and the software for sensor operation was developed with STM32 CubeIDE using C programming language. Operating mode settings include settings for ADC, LED power supply management, and data record frequency. Data recorded with ADC are transmitted to the output device in packets of several kilobytes every several seconds.

The data recording process is not affected by the data transmission. Thereby, data visualization is based on a constant time scale (time delay between two adjacent points). Data reception and visualization programming for the PC (output device) was developed using Python programming language and PyQT library.

### 2.2. Dynamic Light Scattering

To detect the blood flow velocity and analyze the microcirculation state in studied tissues (particularly the finger), the dynamic light-scattering method was used. It is based on detection of not transmitted, but scattered radiation. In the experiment, the scattering intensity depending on time (when no dye was injected) is measured. To process the obtained signals, we calculate the normalized autocorrelation function of the intensity fluctuations of the scattered light. The correlation function in the case of blood microcirculation detection, where the scatterer is erythrocytes in the bloodstream, is described by the following expression [18]:

(13) $$G\left(\tau \right)=e^{-\tau^{2}*\left(\frac{\vartheta^{2}}{\omega^{2}}+\frac{{\left(\omega \pi \sigma \vartheta \right)}^{2}}{{\left(\lambda l\right)}^{2}}\right)}$$

where *ϑ* is the speed of the scatterer, *l* is the distance from the scattering object to the photodetector, λ is wavelength of light from the light source, $\sigma =\frac{l}{\rho +1}$, ρ and ω are z-dependent width and curvature of the wave front of the beam. We denote by *K* the proportionality coefficient determined by the parameters of the optical circuit:

(14) $$K={\left(\frac{1}{\omega^{2}}+\frac{\sigma^{2}}{∆x^{2}}\right)}^{-1/2}$$

where ∆x=λl/πω. Then $\tau_{c}$ is described by the relation:

(15) $$\tau_{c}=\frac{K}{\left|\vartheta \right|}$$

**Figure 6:**
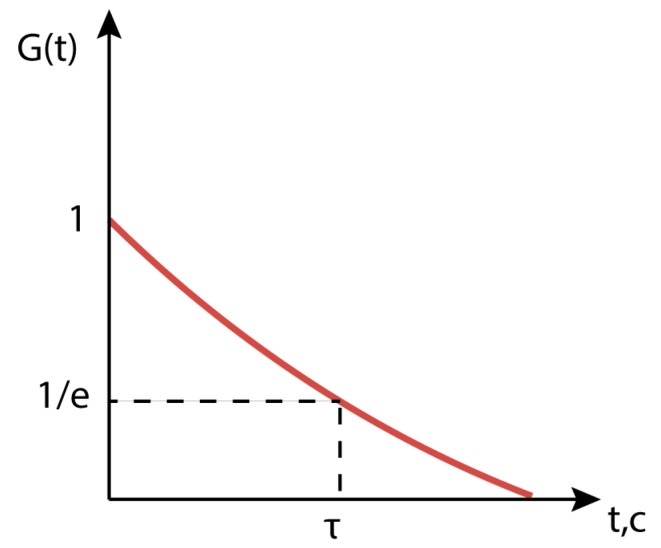
Autocorrelation function of fluctuations in the intensity of radiation scattered by moving objects.

This parameter is called correlation time and is usually determined from the experimental autocorrelation function by the level 1/e of the maximum *G*(*τ*) [19] (Figure 6).

Thus, the expression for the velocity of the scatterer has the form:

(16) $$\left|\vartheta \right|=\frac{1}{\tau_{c}}{\left(\frac{1}{\omega^{2}}+\frac{\sigma^{2}}{∆x^{2}}\right)}^{-\frac{1}{2}}$$

Therefore, the calculation of the autocorrelation function allows us to estimate the average velocity of microcirculatory blood flow in the observation area by the correlation time.

The designed sensor part for registering blood flow velocity includes the following components:

- laser module *λ* = 650 nm, *p* = 5 mW, Δ*f* < 300 GHz, RIN less than −150 dB/Hz (KLM-G650-13-5);
- beam-forming optics (beam diameter in the studied area 1 mm, caustic length 5 mm);
- multimode fiber with a core diameter of 50 µm for scattered light collection and transportation;
- photomultiplier with spectral sensitivity 0.5 · 10^4^ A/W for λ = 650 nm (Hamamatsu H11706-01);
- 14-bit ADC, variable sampling rate up to 50 MHz, input signal range ±10 V (LCard E14-140M).

Laser radiation from a single-mode semiconductor laser passes through the beam forming optics to create a profile of uniform intensity and focus on the finger surface. The wavelength of laser radiation was determined by the characteristics of the absorption spectrum of erythrocytes and the depth of its penetration into tissue. The laser power range was chosen according to the required signal-to-noise ratio, and ranges from 1 to 35 mW. The requirement for radiation monochromaticity is determined by the required coherence length in the scattering volume, in this case ~470.0 ± 0.3 THz. When light hits the skin, part of the radiation penetrates deep into the skin and diffusely scatters on the moving red blood cells. The resulting speckle field is collected by special optics and transferred by multimode fiber to the photomultiplier, which greatly simplifies the detection scheme. The use of fiber avoids the need for a system of lenses, mirrors, and diaphragms to direct radiation to the input aperture of the photomultiplier. In addition, such a scheme makes it possible to reduce the dimensions of the wearable sensor.

**Figure 7:**
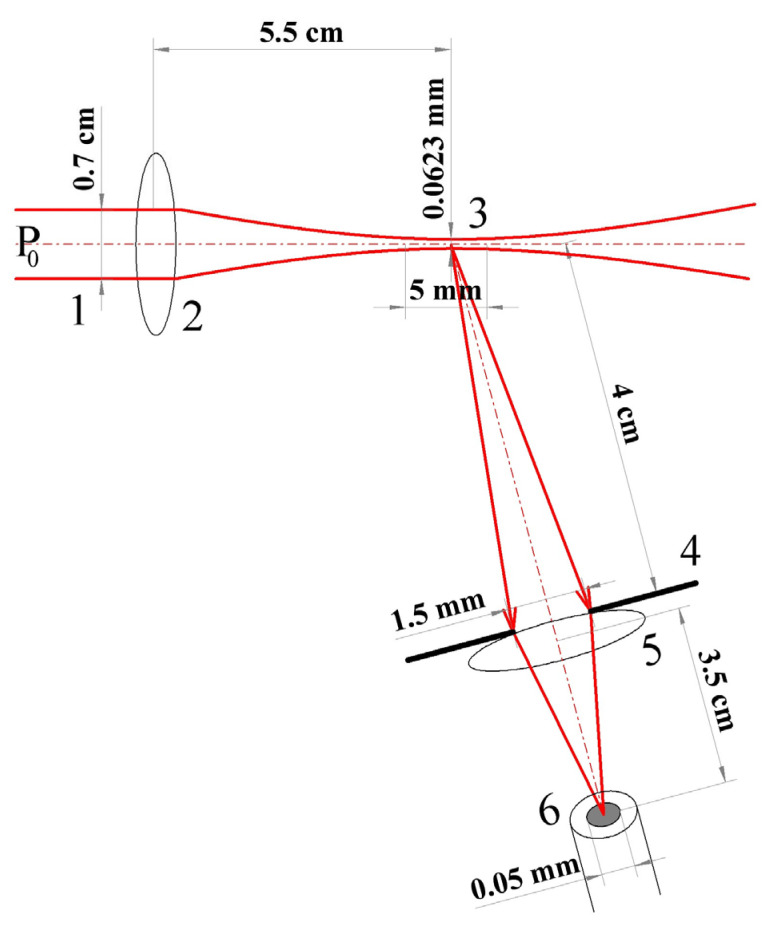
Optical system of registration parts of the measuring system. 1—transmitted beam, 2,5—focusing spherical lens, 3—scattering volume, 4—iris diaphragm, 6—input aperture of the optical fiber.

Registration of the scattered signal is carried out at an angle of 30 degrees. The minimum distance of the location of the recording system from the scattering volume is determined by the condition for the formation of a speckle field in the far zone, >7.5 cm for the parameters of the developed scheme (Figure 7).

The requirements for the sensitivity of the photodetector are determined by the power of the scattering signal, which lies in the range 10^−10^ to 10^−7^ W. The power of the radiation incident on the photodetector and the characteristics of the elements determine the amount of shot noise, which increases inversely with the observation angle of the scattering, while the signal-to-noise ratio decreases.

The signal from the photomultiplier passes to an analog-to-digital converter, where it is amplified, digitized, and then transmitted to a processing unit. The signal recording duration is determined by the typical correlation time (for the samples under study τ_c_ ≈ 1–50 μs) and the required resolution. As the calculation of the correlation function is carried out based on the Wiener–Khinchin theorem using fast Fourier transform of the signal power spectrum, the duration of the signal determines the resolution of the spectrum, and consequently the correlation function. The sampling frequency of the ADC was chosen as 50 kHz according to the Kotelnikov theorem, and the value of the photocurrent spectrum broadening was 1–20 kHz.

The nature of blood flow through the capillary itself is complex: sometimes, against a background of more or less uniform blood flow in the capillary, a short-term slowdown or acceleration of blood flow occurs for 2–4 s, which complicates the analysis of blood flow velocity and makes it difficult to determine its average [20]. Therefore, in order to avoid measurement errors, 10 time samples are recorded during each single experiment.

## 3. Results

### 3.1. Optical Densitometry

**Figure 8:**
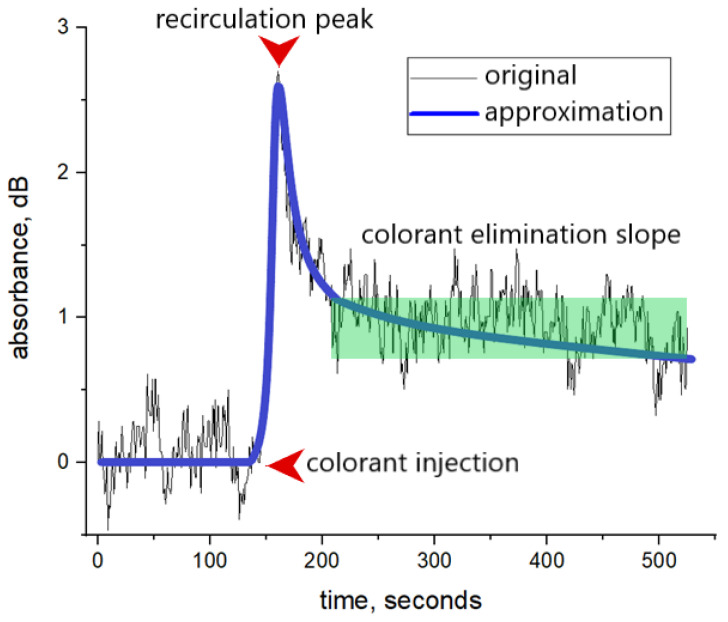
Example of a signal obtained during clearance test and its approximation.

The finger-piece sensor of our design is used to obtain the data on the optical density of the patient’s finger during the routine clearance tests. An example of the obtained signal is presented in Figure 8.

The signal can be divided into three functional periods. The colorant elimination slope contains information about liver metabolic function and can be approximated by Equation (4). The area of the recirculation peak correlates to the cardiac output [21]. Object absorbance function before the colorant injection is averaged to set as the zero point for colorant concentration.

**Figure 9:**
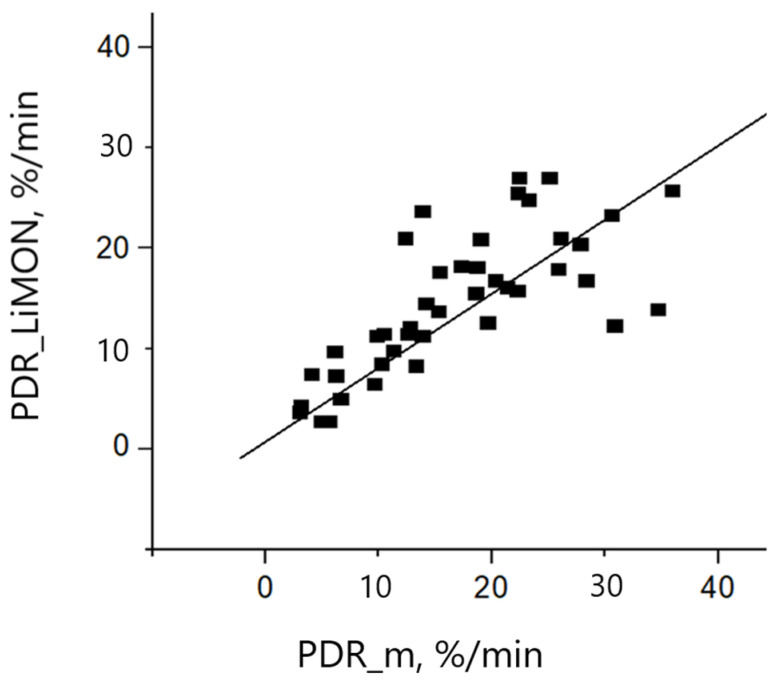
Comparison of PDR measurement by LiMON and our device.

We managed 48 optical densitometry measurements during routine clearance tests to compare our results to LiMON readings. The discrepancies between the measured PDR parameter obtained using the developed sensor and commercial equipment did not exceed 5%/min in most cases. Results of the comparison of PDR calculation results with those obtained using the paper sensor are presented in Figure 9. The correlation coefficient is equal to 0.82, bias is 0.63. (y = 0.63 + 0.82x).

It can be observed that the obtained results are in good accordance. In four of these tests (not presented in the figure) the LiMON measurement resulted in “ERROR”, and no PDR was calculated. For these crucial cases the designed sensor was able to present a PDR value. In three cases the PDR was critically low (<5%/min) and in one case normal liver function with PDR up to 30%/min was diagnosed.

Resolution and accuracy data were also obtained from laboratory tests using liquid phantoms. It was shown that the sensitivity limit of the sensor reached 50 μg/L, which is several orders of magnitude lower than the concentrations used in medical diagnostics.

### 3.2. Dynamic Light Scattering

To check the performance of the developed blood-circulation sensor, test measurements were carried out on model objects. Colloidal light-scattering particles can be used as liquid phantoms. In this research, a solution with microspheres of 1 µm was used as the object of study. The concentration of microspheres corresponds to Gaussian statistics [20]. A solution of microspheres at a given speed flowed through a capillary simulating microcapillaries in tissue. To calibrate the sensor developed according to this scheme and measure the flow rate, the following measurement protocol was developed:

- a solution with a model suspension was prepared;
- a 20 mL syringe was installed into the dispensing apparatus;
- the capillary was fixed, and the volumetric rates set on the dispensing apparatus in the range of 3 to 30 mL/h. Before the direct measurement, a pause was held for several minutes to allow the flow rate of the solution in the capillary simulator to become constant. Before each change in the set speed, the syringe was removed from the dispenser and shaken to avoid stagnation of microspheres in the syringe and capillary;
- the laser was turned on and the program run to record and process light-scattering data;
- the data necessary for measurements were entered into the computer program: the duration of the measurement, the wavelength of laser radiation, the scattering angle, and the name of the experiment;
- a calibration measurement was launched, in which a short light pulse with a duration of 10 ms was applied to the capillary with the test sample and the laser radiation power was adjusted based on the detected scattering intensity, to determine the level of the dark current of the photodetector;
- the scattering signal was recorded as a function of time, with subsequent calculation of the temporal autocorrelation function of light scattering on the sample;
- the received data was processed (calculating the average value of the flow rate, and standard deviation).

**Figure 10:**
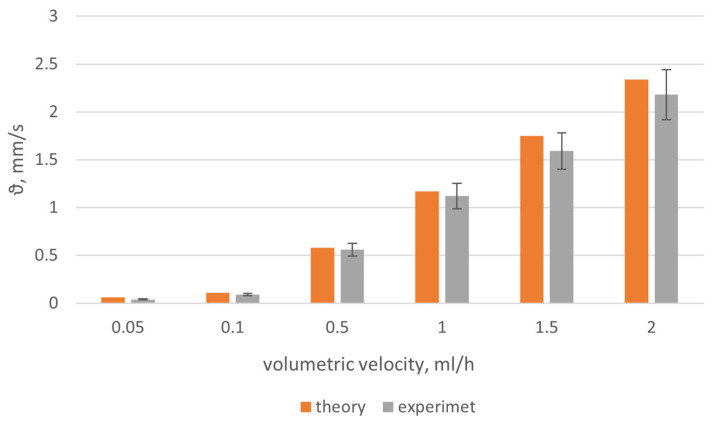
The results of the study of model fluid flow velocity ϑ.

The obtained results for the flow rate of the model fluid ϑ, calculated in accordance with Formula (4), are shown in Figure 10.

The results obtained for a model suspension of particles with a size of 1 μm showed that the sensor parameters and the proposed mathematical apparatus made it possible to obtain correct results. The developed device allowed measurement of the blood flow velocity in the range of 0.05 to 3 mm/s with less than 12% error. The minimum flow velocity that can be measured using the developed sensor is ϑ_min_ = 0.05 mm/s.

**Figure 11:**
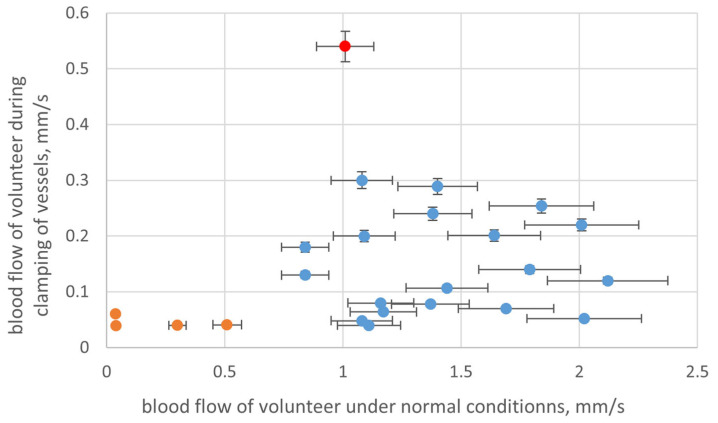
Obtain results for study of blood flow velocity. Blue dots—healthy volunteers, orange dots—volunteers with cardiovascular diseases, red dot—healthy volunteer with abnormally high blood speed (clamped vessel).

After calibration, additional measurements were carried out to test the sensor using volunteers. Participants in the series of experiments were 20 volunteers with no cardiovascular diseases and four volunteers with such diseases. The experiments were carried out in a sitting position, not sooner than 2 h after eating. Before measurement, the pressure and pulse were recorded for each volunteer. The volunteer’s hands were placed on the table at the level of the heart. The microcirculation rate was recorded for 1 min, with the sensor fixed on the surface of the distal phalanx of the finger. Figure 11 presents the results of the study for 24 volunteers. The experiment was conducted under two different conditions: normal conditions, and with clamping of vessels.

Microcirculatory blood flow of these skin areas is normally regulated to a greater extent by sympathetic mechanisms due to the presence of arteriovenous anastomoses. For a healthy person, this rate lies between 0.5 and 3 mm/s. A similar trend was detected in our study (Figure 11, blue). In addition, high variation in blood flow velocity between normal and clamped vessel states was detected for healthy volunteers. Significantly lower variation and microcirculation speed in general was detected for people with cardiovascular diseases.

## 4. Discussion

The PDR coefficients detected with the use of the designed sensor system in the present study are in good accordance with commercial sensors, demonstrating the reliability of the analyzed data. Furthermore, in several cases, we demonstrated a crucial advantage of the presented sensor which is its ability to detect correct results in edge cases. From a total of 48 conducted measurements, four were not analyzed by the LiMON sensor but were processed by the designed sensor system. Such situations frequently appear in medical practice and can now be avoided.

The obtained results for blood microcirculation speed in healthy volunteers showed normal variation in the range from 0.8 to 2.5 mm/s, which is in good accordance with known data. For volunteers with cardiovascular diseases, the rate of blood flow velocity varied from 0.1 to 0.5 mm/s, which clearly indicates specific microcirculation disorders. Comparison of blood flow speed in normal and clamped vessels can specify the vessels’ tonus. Figure 9 shows one abnormal point (in red) where there was low variation in the blood flow velocity of normal and clamped vessels. Detailed analysis of volunteers’ history showed that the person behind the red dot was a professional sports player. Low variation, in this case, may be the result of linear velocity of blood flow depending on the tone of microvessels, myogenic, neurogenic, and endothelial factors of microcirculation, and on changes in passive factors (pulse wave, the action of the “respiratory pump”). The magnitude of the linear velocity of capillary blood flow can be formed at the level of larger and deeper vessels located in muscle-containing arterioles. An increase in their tone can lead to an increase in the linear velocity of blood flow in the capillaries of the skin. This is characteristic mainly of the conditions of vasodilation, with increased perfusion, which is often observed in younger people in good physical condition.

Thus, using the method of dynamic light scattering, clinicians can draw conclusions about the presence of various kinds of diseases by assessing the quantitative values of peripheral blood flow. Many studies have shown that changes in the microcapillary bloodstream occur in patients with hepatitis of the liver with moderate or high histological activity and fibrosis or cirrhosis of the liver [22,23]. It has been shown that in these cases, the blood flow velocity in the microvasculature can decrease to 0.05 mm/s. Therefore, the combined sensor design will allow specification of the decompensation of diffuse liver diseases in terms of extrahepatic manifestations by assessing the quantitative values of peripheral blood flow (perfusion, concentration, and speed).

The noise amplitude observed in the experiment with dye elimination and blood microcirculation speed clearly significantly exceeded the theoretical estimates of minimum error. More importantly, the noise in the non-invasive measurement exceeded the noise power in the simulated experiment. This difference suggests that the method can be improved by additional analysis of noise sources. The light that enters the receiver after passing through the digit (finger) contains information about the movement of the finger, as well as the movement of substances inside the finger, primarily capillary blood flow. In addition, the rate of dye removal from the blood depends not only on the metabolic activity of liver cells but also on the amount of blood passing through the liver per time unit.

## 5. Conclusions

Evaluation of the metabolic function of the liver by analyzing the dynamics of the dye removal from the blood is of great importance for diagnosis, including for predicting the consequences of surgery. Non-invasive analysis of dye excretion dynamics is faster and safer than invasive methods, and the number of non-invasive measurements in one test is not limited as with blood samples. However, there are significant interferences that may affect the results of non-invasive measurements.

In the presented research, a laboratory model of a combined sensor for optical densitometry and dynamic light scattering was created and software was developed to control the operation of the sensor and process the recorded data. The resolution of the sensor was calculated when registering the flow rate of a solution of microspheres in a simulation of human microcapillaries at the concentration of ICG in the human body. Calibration and modeling experiments were carried out to adjust and calibrate the sensor and software. The error in determining the flow rate of microspheres was about 10%. The minimum value of the flow rate was ϑ_min_ = 0.05 mm/s.

Testing of the sensor in medical practice demonstrated its high reliability and ability to analyze edge cases, which is a crucial advantage in diagnostics. Inclusion of the blood microcirculation velocity in liver function diagnostics is a promising approach, although statistical studies demand to analysis of the specific connection between microcirculation and PDR parameters [24,25]. In the future, it is planned to develop a neural network system for medical analysis of the data obtained to reveal this connection and as a result provide accurate diagnosis, a comprehensive assessment of liver function, and prediction of the further course of the disease, as well as to predict the success of surgical intervention. In this case, the input parameters of the neural network will be the parameters measured by the joint sensor system: pulse, oxygenation level, elimination rate and level of residual concentration of ICG, and blood flow velocity in the microvasculature. To create a neural network medical classifier, we will use a system architecture that implements the approach of training an artificial neural network with an “interval teacher” [26]. Prediction of the patient’s condition will be implemented using a trained intelligent neural network module [27] based on the data collected during the examination of the patient.

## Data Availability

Not applicable.
